# Comparison of two surgical methods for the treatment of CIN: classical LLETZ (large-loop excision of the transformation zone) versus isolated resection of the colposcopic apparent lesion – study protocol for a randomized controlled trial

**DOI:** 10.1186/s13063-015-0736-8

**Published:** 2015-05-23

**Authors:** Theresa M. Schwarz, Thomas Kolben, Julia Gallwas, Alexander Crispin, Christian Dannecker

**Affiliations:** Department of Obstetrics and Gynaecology, Ludwig Maximilian University, Campus Grosshadern, Munich, D-81377 Germany

**Keywords:** Cervical dysplasia, Cervical intraepithelial neoplasia, CIN, Conization, HPV, LEEP, LLETZ, Transformation zone

## Abstract

**Background:**

In compliance with national and international guidelines, non-pregnant women with cervical intraepithelial neoplasia grade 3 should be treated by cervical conization. According to the definition of the large loop excision of the transformation zone (LLETZ) operation, the lesion needs to be resected, including the transformation zone. It is well known from the literature that the cone size directly correlates with the risk of preterm delivery in the course of a future pregnancy. Thus, it would be highly desirable to keep the cone dimension as small as possible while maintaining the same level of oncological safety.

**Methods/Design:**

The aim of this study is to analyze whether resection of the lesion only, without additional excision of the transformation zone, is equally as effective as the classical LLETZ operation regarding oncological outcome. We are performing this prospective, patient-blinded multicenter trial by randomly assigning women who need to undergo a LLETZ operation for cervical intraepithelial neoplasia grade 3 to either of the following two groups at a ratio of 1:1: (1) additional resection of the transformation zone or (2) resection of the lesion only. To evaluate equal oncological outcome, we are performing human papillomavirus (HPV) tests 6 and 12 months postoperatively. The study is designed to consider the lesion-only operation as oncologically not inferior if the rate of HPV high-risk test results is not higher than 5 % compared with the HPV high-risk rate of women undergoing the classical LLETZ operation.

**Discussion:**

In case that non-inferiority of the “lesion-only” method can be demonstrated, this operation should eventually become standard treatment for all women at childbearing age due to the reduction in risk of preterm delivery.

**Trial registration:**

German Clinical Trials Register (DRKS) Identifier: DRKS00006169. Date of registration: 30 July 2014.

## Background

Cervical cancer is the third most common cancer diagnosis and the fourth-leading cause of death in women worldwide, responsible for 9 % (529,800) of all new cancer cases and 8 % (275,100) of deaths caused by cancer in women in 2008 [1].

Precursor lesions of squamous cell carcinoma of the cervix uteri, which represent about 80 % of cervical cancers, are referred to as *cervical intraepithelial neoplasia* (CIN), which is distinguished in three degrees of severity (CIN 1 to 3) [2]. Compared with invasive cervical cancer, the occurrence of precancerous lesions of the cervix uteri is much higher.

A prerequisite for the development of cervical dysplasia is a persistent infection with high-risk human papillomavirus (HPV) types [3]. Anogenital HPV infection is a common sexually transmitted disease, estimated to affect 75 % to 80 % of sexually active adults younger than age 50 years [4]. The majority of HPV infections are self-limiting within 1 to 2 years [5]. These high-risk HPV types, particularly HPV 16 and 18, are responsible for the vast majority of cervical cancers [6], whereas low-risk HPV types are associated with condylomata acuminata.

HPV infections affect undifferentiated basal cells of the cervical epithelium within the transformation zone. The transformation zone is the area of the cervix where the columnar epithelium of the endocervix meets the squamous epithelium of the ectocervix.

Before puberty, the squamocolumnar junction lies within the endocervical canal. Owing to hormonal changes during puberty, the columnar epithelium extends outward and the squamocolumnar junction moves onto the ectocervix. Under exposure to the acidic vaginal environment, columnar epithelium can undergo physiological metaplasia and transform into squamous epithelium. The new squamocolumnar junction is then closer to the endocervix compared with the original squamocolumnar junction, and the zone in between is called the *transformation zone* of the cervix. Recently, a hypothesis has been stated that cervical cancer emerges from a newly described embryological cell population. These so-called cuboid cells are located directly at the squamocolumnar junction [7].

In accordance with national and international guidelines [8], standard treatment of non-pregnant women diagnosed with CIN 3 is conization of the cervix. This procedure is also called *large loop excision of the transformation zone* (LLETZ). The rationale for the removal of the transformation zone in addition to the lesion itself is that both the precursor lesion and cervical cancer itself typically arise in the area of the transformation zone.

In addition to general complications such as intraoperative or postoperative bleeding, infection, pain and scarring, cervical conization is associated with a significantly high rate of cervical weakness and a consequent increase in miscarriages and premature birth rates in future pregnancies [9–12]. This is of special interest because patients diagnosed with cervical dysplasia are typically of childbearing age, with an average age of 30 years [13, 14]. It is well known that the risk of preterm birth is directly correlated with the size of the removed cone [15–17]. With knowledge of these serious complications, there is a worldwide trend aimed at reducing surgical radicalism by removing the dysplastic lesion only, without additional resection of the transformation zone.

In a large retrospective study of over 150,000 women treated for CIN 3 between 1958 and 2008, it has lately been shown that women who received treatment more recently were at greater risk of developing cervical cancer [18]. It is suggested that this observation directly correlates with the use of less aggressive treatment over the last two decades [19].

With awareness of this development, it is important to respecify the necessary extent of a conization. Primarily, the optimum cone size needs to guarantee the highest oncological safety defined by low recurrence and high R0 resection rates. Simultaneously, the extent of the resection should be as small as possible to reduce the morbidity risk during subsequent pregnancies.

The primary objective of the present study is therefore to compare the classical LLETZ operation (that is, resection of the lesion with entrainment of the transformation zone) with resection of the colposcopically abnormal lesion only in the light of oncological safety.

## Methods/Design

### Study design

The Evaluation of Clinical Outcome after Reduction of Conization Size (or ECO-ROCS) trial is a multicenter, prospective, parallel-group, patient-blinded, non-inferiority randomized controlled trial in which we are assessing oncological comparability of two conization procedures: (1) classical LLETZ, comprising excision of the dysplastic lesion, including resection of the transformation zone; and (2) excision of the dysplastic lesion only.

### Study setting

This study is being conducted in 13 study centers, all of which run a specialized dysplasia clinic (Munich (two), Hannover, Wolfsburg, Berlin, Kiel, Regensburg, Stralsund, Hamburg, Düsseldorf, Aachen, Ulm, Münster). The participating study centers and investigators are fully listed in the German Clinical Trials Register (DRKS00006169).

### Sample size and statistics

A sample size of 892 patients was calculated with the POWER procedure of SAS for Linux 9.3 software (SAS Institute, Cary, NC, USA) using the following parameters: non-inferiority margin of 5 %, α = 0.05, 80 % power and one-sided test. A rate of 90 % negative HPV test results 6 months after conization was taken as a reference [20] for both groups. Assuming a 10 % dropout rate, we aim for a sample size of 1000 patients.

The statistical inference regarding the primary outcome will be based on a one-sided 95 % confidence interval for the risk difference, calculated as the proportion of HPV-positive patients in the control group minus the corresponding proportion in the experimental group. If the upper limit of the confidence interval is less than the non-inferiority margin of minus 5 %, non-inferiority will be regarded as established.

### Recruitment und randomization

Patients are being seen and diagnosed in specialized dysplasia outpatient clinics of the participating study centers. After they provide us their written informed consent, participants fulfilling all required inclusion and exclusion criteria are given a five-digit pseudonym (patient identifier), with the first two numbers indicating the study center and the latter three numbers indicating a sequential patient number. Patients are randomly assigned by their pseudonym with equal probability to either of the two treatment arms, using a 1:1 ratio. Randomization is carried out using the web-based Randoulette randomization service of the Institute for Medical Informatics, Biometry and Epidemiology, University of Munich, Germany. The platform offers a web-based form in which to enter patient identifier, sex, age and study center, as well as the strata of the patient. Access to the randomization service is provided to the two leading investigators at each study center. Patients are stratified by age (>30 or ≤30 years) and study center.

After the information is entered, the study arm is selected according to a predefined randomization scheme, in this case an unblended, stratified block randomization. To maintain allocation concealment, the exact length of blocks is kept secret by the Randoulette randomization service. The entered information, as well as the randomization results, are stored in the Randoulette database.

### Ethical approval

Approval of trial protocol and informed consent documents have been obtained primarily from the University of Munich Institutional Review Board (project number 275-14), followed by the responsible ethics committees and/or state chambers of physicians of the 12 other study centers (University of Munich Institutional Review Board, ethics committee of the Medical Chamber of Westphalia-Lippe, University of Regensburg Institutional Review Board, ethics committee of the Medical Chamber of Hamburg, ethics committee of the Hannover Medical School, State Medical Chamber of Lower Saxony, institutional review board of Berlin, ethics committee of the Medical Chamber of Schleswig-Holstein, ethics committee of the Heinrich-Heine University of Düsseldorf, institutional review board of Greifswald, University of Aachen Institutional Review Board and institutional review board of the University of Ulm).

All study staff commit themselves to the Declaration of Helsinki (2013 version), as well as to all pertinent national laws and the International Conference on Harmonisation of Technical Requirements for Registration of Pharmaceuticals for Human Use (ICH) harmonized tripartite guidelines for Good Clinical Practice issued in June 1996 (http://www.ich.org/fileadmin/Public_Web_Site/ICH_Products/Guidelines/Efficacy/E6/E6_R1_Guideline.pdf) and the European Medicines Agency’s CPMP/ICH/135/95 guideline (July 2002; http://www.edctp.org/fileadmin/documents/EMEA_ICH-GCP_Guidelines_July_2002.pdf). Important protocol modifications will be reported to all relevant parties. All participants complete informed consent forms prior to participation in the trial.

### Inclusion and exclusion criteria

#### Inclusion criteria

Patients with an internal or external diagnosis of histologically confirmed CIN 3, a colposcopically visible lesion and a positive high-risk HPV test (according to the criteria of Meijer *et al*. [21]) who have had no previous cervical surgery, no previous treatment of the disease, are at least 18 years of age and provide written informed consent are eligible for inclusion in the study.

#### Exclusion criteria

Patients are excluded at any time upon their request or if any of the following applies: pregnancy at time of inclusion and during the first 6 postoperative months, intake of immunosuppressants, known HIV infection, malignant disease requiring treatment or inadequate colposcopy.

### Study blinding

To exclude bias by unconsciously different behavior, the study is patient-blinded for the first 6 postoperative months. In case of complications, however, access to the surgical report is possible at any time.

### Surgery

**Fig. 1:**
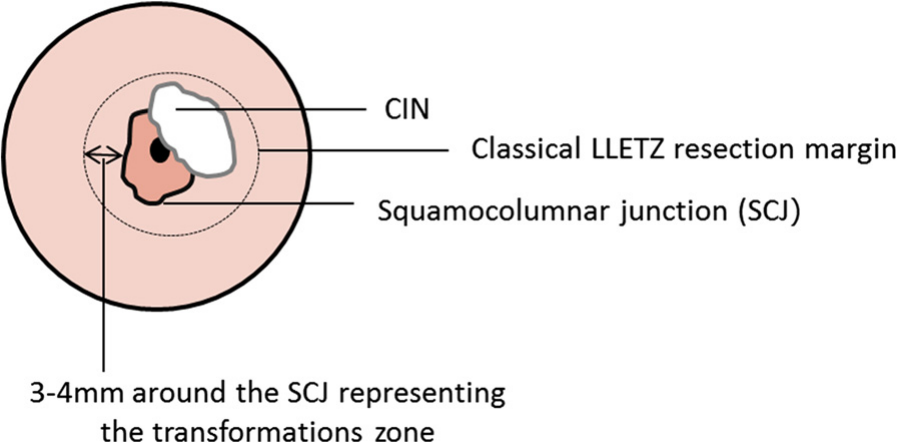
Resection margins of classical large loop excision of the transformation zone operation including the transformation zone, which is defined as 3 to 4 mm around the squamocolumnar junction. CIN, Cervical intraepithelial neoplasia; LLETZ, Large loop excision of the transformation zone; SCJ, Squamocolumnar junction

**Fig. 2:**
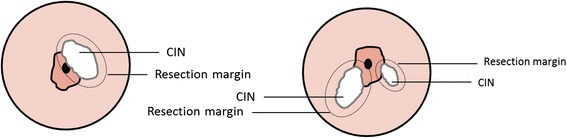
Resection margins in lesion-only operation, where a distance of 2 mm around the lesion should be kept. CIN, Cervical intraepithelial neoplasia

**Fig. 3:**
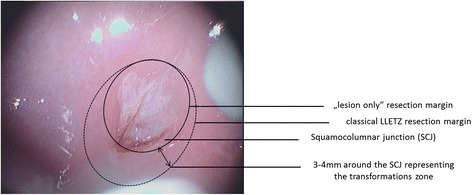
Comparison of the extent of both surgical methods, example 1. LLETZ, Large loop excision of the transformation zone; SCJ, Squamocolumnar junction

**Fig. 4:**
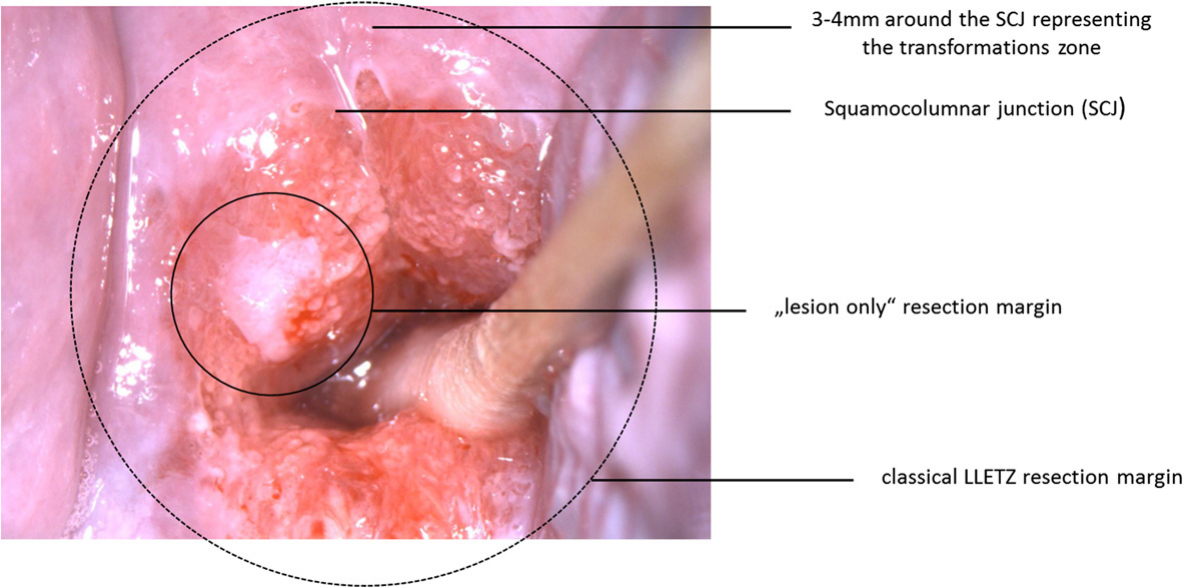
Comparison of the extent of both surgical methods, example 2. LLETZ, Large loop excision of the transformation zone; SCJ, Squamocolumnar junction

Both surgical techniques are performed using a thin, low-voltage, electrified wire loop known as a *loop electrosurgical excision procedure electrode*. Procedures are performed by experienced surgeons at 13 German dysplasia centers under colposcopic control and are carried out as shown in Figs. 1, 2, 3 and 4. A dysplastic cervical lesion is identified after application of acetic acid and iodine solution.

In the lesion-only resection, the dysplastic area is removed with a safety margin of 2 mm. In the classical LLETZ, the lesion, including the transformation zone, is resected. Because the outer border of the transformation zone cannot be determined colposcopically, the following definition for resection of the transformation zone was stated after expert consensus. After identification of the squamocolumnar junction, an area of 4 mm surrounding the junction is defined as the transformation zone. In the event that the iodine-negative zone is greater than the defined area, the iodine-negative area is considered the transformation zone and should be removed as well.

Postoperatively, cone size is measured before fixation by submergence in a fluid-filled cylindrical vial using Archimedes’ principle. Afterward the specimen is evaluated histologically for the grade of dysplasia and achievement of resection margins.

### Postoperative controls

According to German guidelines, the first follow-up examination is performed after an interval of 6 months. A Pap smear, HPV test and colposcopy are performed. In cases of colposcopically suspect lesions, a biopsy is taken. A second postoperative control is carried out 12 months after conization.

### Primary outcome measure

The primary endpoint is the rate of negative high-risk HPV tests after 6 months, because a negative HPV test is generally regarded as evidence of successful treatment. The negative predictive value for a negative HPV test after conization is between 92 % [16] and 100 % [22–26], and successful treatment usually leads to the elimination of the virus [27–30]. A negative HPV test therefore excludes CIN persistence or recurrence with high probability. In contrast, repeated positive HPV tests can be taken as suspicious of a continuation of dysplasia or as an indicator of recurrent dysplasia.

### Secondary outcome measure

Because it is possible, from a clinical point of view, that patients who have received the lesion-only operation simply need more time for the viral infection to be eliminated, HPV is tested again in the postoperative control after 12 months. Further secondary endpoints are Pap smear results in both postoperative controls, biopsy results (if applicable) and resection margins.

### Safety concerns

It should be noted that, even with classical conization, resection margins are positive or only questionably negative in up to 25 % of cases.

If there is CIN 1 detectable at the cutting margins, recurrence or persistence is expected in only 0 % to 5 % of participants. In the case of CIN 3 at the surgical margins, recurrence can be expected in 20 % to 25 %. If non in sano resection happens at the endocervical margin, risk increases to 30 %. If both, endo- and ectocervical resection margins are affected with CIN 3, risk is highest with 50 % [31]. Importantly, in those cases where immediate reconization is indicated because of positive surgical margins, no dysplasia was detected in over 80 % of cases.

For this reason, an immediate reconization usually is not indicated, but should be assessed according to individual risk and discussed with the patient. Only after further histological confirmation of CIN during the first postoperative control after 6 months will reconization be recommended.

Thus, the initially conservative approach in terms of a checkup after 6 months is also justified in the case of an increased incidence of positive surgical margins. This can be seen especially in light of the expected reduction of complications associated with surgery.

### Interim analysis and stopping rules

An interim analysis will be performed at the postoperative 6-month control of the first 250 patients included. The primary outcome is the result of HPV tests. If the rate of positive HPV tests after 6 months in patients who have undergone lesion-only surgery is higher by more than 15 % than among patients who have had classical LLETZ, the study will be terminated. Because the interim analysis was included exclusively for safety reasons and thus does not involve a test of the non-inferiority study hypothesis, the probability of a type I error (that is, a false-positive study result) should not be affected.

### Additional investigations

Because HPV infection is the cause of dysplasia, further analyses to investigate HPV infection are performed in a subset of 250 patients. A particular focus is on the genotyping of HPV infection because often multiple infections with different HPV types are present simultaneously, especially in young patients. Thus, there is the risk that, despite eradication of the lesion-causing HPV type, the conventional HPV test result may continue to be positive because infection with another HPV type is present without clinical consequence.

To make a statement with respect to a more valid clinical consequence of HPV infection, detection of transcriptional activity of E6 and E7 is analyzed. Expression of viral oncoproteins E6 and E7 plays a crucial role in HPV-mediated carcinogenesis, as it interferes with the cell cycle, causes cell cycle progression (E7) and at the same time prevents apoptosis (E6). This occurs through the inactivation of oncosuppressor proteins p53 and retinoblastoma protein (pRb). pRb in particular prevents cells from entering S-phase and thus has antiproliferative effects [32–37]. As opposed to the mere detection of HPV DNA, testing for the transcriptional activity of E6 and E7 can allow us to make a statement about the biological activity of the infection.

The described additional analyses are performed at five study centers (Munich, Hannover, Wolfsburg, Berlin and Regensburg).

## Discussion

With knowledge about the obstetric complications of cervical conization, surgical radicalism has increasingly declined in recent years. To date, it has not yet been systematically analyzed whether consistent oncological safety can be guaranteed by removing less tissue. In discussions about the optimal extent of conization with leading colposcopists in Germany, it furthermore turns out that the size of the cone varies significantly, depending on the individual surgeon.

The aim of this study is thus to define a universal standard for cervical conization that combines the highest level of oncological safety with minimal obstetric risk. To that end, we compare two surgical methods with each other: (1) classical LLETZ, in which the lesion is removed, including the transformation zone; and (2) excision of the lesion only.

There are certain limitations to our study. In patients in whom the entire transformation zone is affected by the lesion but who will be randomized into the lesion-only group, the extent of the operation would in fact be the same as in patients randomized to undergo the classical LLETZ operation. This could ultimately lead to limited discrimination between the two study groups. To evaluate this influence on the results, every surgeon must indicate the extent of the dysplastic area (CIN/ectocervix in percent) at the time of the operation. However, including only patients with small lesions would lead to a severe selection bias by recruiting patients according to more subjective criteria. Furthermore, generalization of the study results could no longer be guaranteed, because only a subgroup of patients with high-grade dysplastic lesions would be included.

## Trial status

As of May 2015, we are still recruiting participants.

## Abbreviations

CIN: Cervical intraepithelial neoplasia
ECO-ROCS: Evaluation of Clinical Outcome after Reduction of Conization Size trial
HPV: Human papillomavirus
ICH: International Conference on Harmonisation of Technical Requirements for Registration of Pharmaceuticals for Human Use
LEEP: Loop electrosurgical excision procedure
LLETZ: Large loop excision of the transformation zone
pRb: Retinoblastoma protein
SCJ: Squamocolumnar junction
